# Composition and biosynthetic machinery of the *Blumeria graminis* f. sp. *hordei* conidia cell wall

**DOI:** 10.1016/j.tcsw.2019.100029

**Published:** 2019-08-14

**Authors:** Trang A.T. Pham, Bianca A. Kyriacou, Julian G. Schwerdt, Neil J. Shirley, Xiaohui Xing, Vincent Bulone, Alan Little

**Affiliations:** a ARC Centre of Excellence in Plant Cell Walls, School of Agriculture, Food and Wine, University of Adelaide, Waite Campus, Glen Osmond, SA 5064, Australia; b Adelaide Glycomics, School of Agriculture, Food and Wine, University of Adelaide, Waite Campus, Glen Osmond, SA 5064, Australia

**Keywords:** *Blumeria graminis* f. sp. *hordei*, Cell wall, Glycosyltransferase, Glycoside hydrolase

## Abstract

Infection of barley with the powdery mildew causal agent, *Blumeria graminis* f. sp. *hordei* (*Bgh*), can lead to devastating damage to barley crops. The recent emergence of fungicide resistance imposes a need to develop new antifungal strategies. The enzymes involved in cell wall biosynthesis are ideal targets for the development of fungicides. However, in order to narrow down any target proteins involved in cell wall formation, a greater understanding of the cell wall structure and composition is required. Here, we present a detailed carbohydrate analysis of the *Bgh* conidial cell wall, a full annotation of Carbohydrate Active enZymes (CAZy) in the *Bgh* genome, and a comprehensive expression profile of the genes involved in cell wall metabolism. Glycosidic linkage analysis has revealed that the cell wall polysaccharide fraction of *Bgh* conidia predominantly consists of glucosyl residues (63.1%) and has a greater proportion of galactopyranosyl residues compared to other species (8.5%). Trace amounts of xylosyl residues were also detected, which is unusual in ascomycetes. Transcripts of the genes involved in cell wall metabolism show high expression of chitin deacetylases, which assist fungi in evading the host defence system by deacetylating chitin to chitosan. The data presented suggest that the cell wall components of the conidia and the corresponding obligate biotrophic CAZy gene profile play a key role in the infection process.

## Introduction

Plant pathogens present significant constraints on the yield and quality of agricultural crops. Powdery mildew infestations in barley are caused by *Blumeria graminis* f. sp. *hordei* (*Bgh*), an obligate biotrophic airborne fungus. In many parts of the world powdery mildew has emerged as one of the most economically important barley diseases, as a single outbreak alone can cause up to 20–40% yield loss (Akhkha, 2009, Murray and Brennan, 2010).

One of the most common strategies to control fungal pathogens is by breeding disease resistant cultivars that can specifically detect the pathogen and avoid infection. However, the resistance is usually monogenic and the fungi are eventually able to circumvent the resistant genes within a few years (Wolfe and Mcdermott, 1994). While disease resistant breeding is a viable strategy it is a time-consuming process and may not even benefit in the long run as some resistant cultivars have shown reductions in yield performance. In conjunction with breeding resistant cultivars, an arsenal of fungicides are also used to control disease outbreaks. However, powdery mildew has a propensity towards rapidly developing resistance to the various fungicides. Potential fungicides that could be deployed in the future may not be viable if *Bgh* can overcome their mode of action (Reis et al., 2013, Tucker et al., 2015). The emergence of fungicide-resistant strains is a global issue necessitating the need for new antifungal strategies.

Fungal cells are encompassed in a complex network of interconnected polysaccharides and proteins known as the cell wall (Free, 2013, Gow et al., 2017). As the first point of contact between the fungus and the environment, the fungal cell wall influences the interactions with other organisms and holds a critical role in determining if the environmental conditions are hostile or favourable. The cell wall protects the fungal cell against all manners of environmental adversities, i.e. from desiccation, extreme temperatures, pH changes, oxidative agents, and mechanical injuries (Levin, 2011). In the case of pathogenic fungi, additional stresses may arise from the host defence responses and antifungal agents. The dynamic nature of the cell wall makes it essential during morphogenesis, cell viability, and pathogenesis (Durán and Nombela, 2004). In addition, a significant part of the genome, e.g. 20% in the case of the yeast *Saccharomyces cerevisiae*, is dedicated to cell wall metabolism, which further reflects the importance of the fungal cell wall (de Groot et al., 2001).

In phytopathogenic fungi the cell wall is critical for successful penetration of the host. The infection structures that the fungi develop are the focal point for enzymatic activity and increased turgor pressure is required for the fungal penetration peg to breach the plant cell wall. It is therefore prudent to disrupt the biosynthesis of the fungal cell wall before it can facilitate these functions. The fungal cell wall contains vital polysaccharides that are not present within the cell walls of plants. This makes the enzymes involved in their biosynthesis ideal targets for antifungal drugs and disease control.

Fundamentally the overall cell wall structure and architecture is conserved across all fungal species (Coronado et al., 2007). However, while many of the underlying building blocks are conserved other components are species-specific or even morphologically-specific. The cell wall core consists of a conserved network of chitin and β-1,3-glucans along with an outer layer of heterogeneous glycoproteins. The chitin and β-1,3-glucans interconnect and assemble into a load bearing structural scaffold for the glycoproteins (Klis et al., 2006).

The structure and composition of the oligosaccharide chains attached to the glycoproteins vary significantly between fungi. While many of these glycans are composed of mannosyl residues, they can also comprise a multitude of other sugars such as glucose, galactose and fucose (Free, 2013). Yeast, such as *S. cerevisiae* and *Candida albicans*, possess an outer layer comprised of highly mannosylated glycoproteins (Ruiz-Herrera et al., 2006, Orlean, 2012) whereas the filamentous fungi *Neurospora crassa* and *Aspergillus fumigatus* have an outer layer of galactomannans (Fontaine et al., 2000, Free, 2013). Collectively the glycoproteins generate a chemically inert shield that limits the permeability of the cell wall, defending the internal framework of polysaccharides from degradation (Erwig and Gow, 2016).

To successfully design new and efficient fungal cell wall inhibitors, a finer understanding of cell wall polysaccharides and their biosynthesis in phytopathogenic fungi is required. To acquire insight into the cell wall composition of *Bgh* we analysed the polysaccharide composition of ungerminated conidia. In addition, we examined the Carbohydrate-Active enZymes (CAZy) encoded by the *Bgh* genome and investigated the expression profile of cell wall polysaccharide biosynthetic and hydrolytic genes in the ungerminated *Bgh* conidia.

## Materials and methods

### Fungal material

A field isolate of *Bgh*, kindly donated by Professor Richard Oliver (Curtin University, W.A., Australia), was maintained on the highly susceptible barley cultivar Baudin. *Bgh* was propagated by inoculating seven-day-old barley seedlings with conidia from previously infected barley seedlings. The inoculated plants were then cultivated in a growth chamber with a 16 h photoperiod at 22 °C for a week. *Bgh* conidia were collected by vacuuming them directly from the plants through a 35 μm filter and capturing them on a 5 μm filter.

### Preparation of cell walls

The fungal sample was snap frozen in liquid nitrogen and freeze-dried. Alcohol insoluble residue (AIR) was prepared from the *Bgh* conidia as previously described (Mélida et al., 2013). The AIR was then de-starched using α-amylase, recovered by ethanol precipitation, and dehydrated under vacuum as described by Pettolino et al. (2012). The AIR was stored in a desiccator until required.

### Glycosidic linkage analysis

The cell wall sample was treated with acetic anhydride in aqueous, methanolic acetic acid solution to per-*N*-acetylate amino sugars (Hirano et al., 1976). Uronic acids were converted to their 6,6-dideuterio neutral sugar counterparts using carbodiimide activation at pH 4.75 followed by reduction with sodium borodeuteride (NaBD_4_) at pH 7.0 (Kim and Carpita, 1992). All carbohydrate polymers present in the sample were permethylated (Ciucanu and Kerek, 1984), hydrolysed with trifluoroacetic acid (TFA), reduced with NaBD_4_, and peracetylated with acetic anhydride to generate partially methylated alditol acetates (PMAAs) (Pettolino et al., 2012). The PMAAs from neutral sugars and amino sugars were separated by gas chromatography as described earlier (Mélida et al., 2013, Mélida et al., 2015). An Agilent J&W VF-23 ms capillary column (30 m × 0.25 mm i.d.) and an Agilent J&W DB-1 ms Ultra Inert (UI) capillary column (30 m × 0.25 mm i.d.) were used for the separation of PMAAs from neutral and amino sugars, respectively, on an Agilent 7890B/5977B GC–MS system coupled to a flame ionization detector (FID). The PMAAs were then identified by comparing their MS fragmentation patterns with those of reference derivatives and by referring to the literature (Carpita and Shea, 1988), and quantified based on the FID response (Sweet et al., 1975, Carpita and Shea, 1988). Five separate experiments were conducted.

### RNA extraction and RNA-Seq library creation

One hundred mg of *Bgh* conidia was ground in liquid nitrogen with a mortar and pestle. Total RNA was extracted using the Plant Total RNA Kit (Sigma) as per the manufacturer’s instructions. RNA-Seq and library preparation were performed at the Australian Genome Research Facility (AGRF Ltd., Melbourne, Australia). Libraries for three independent biological replicates were prepared using the Illumina Stranded mRNA kit and 100-bp single-ended sequence reads collected using a Illumina HiSeq sequencer. Each run produced up to 20 million reads per biological replicate.

For each of the samples, sequence reads were quality trimmed and reads were mapped to the *Bgh* DH14 BluGen ( www.blugen.org/) genome assembly version 3.0 based on BluGen gene models from July 2012 (gff-file can be obtained via www.mpipz.mpg.de/23693/Powdery_Mildews) using the CLC Genomics Workbench v8.0 (CLC bio, Aarhus, Denmark). Mean gene expression from each sample was expressed as reads per kilobase of transcript per million mapped reads (RPKM). Sequence read data will become available at the NCBI sequence read archive (PRJNA55765) upon publication.

### Quantitative polymerase chain (qPCR) reaction validation

To confirm the reliability of transcript levels, the relative expression levels of 13 cell wall related genes were verified by qPCR. cDNA was synthesised from total RNA using the SuperScript® III Reverse Transcriptase (RT) enzyme from Life Technologies (Carlsbad, U.S.A.) following the manufacturer’s protocol. Transcript profiling was carried out using qPCR as described in Burton et al. (2010) using the gene-specific primers listed in Supplementary Table S1. The data were normalised against the geometric mean of four *Bgh* housekeeping genes, namely actin (*bgh00992*), glyceraldehyde 3-phosphate dehydrogenase (*bgh00075*), tubulin (*bgh01972*), and ubiquitin (*bgh03777*).

### Transcriptome analysis and genome annotation

Kyoto Encyclopedia of Genes and Genomes (KEGG), Enzyme Commission (EC) and PFAM annotations were retrieved from the EnsemblFungi website ( http://fungi.ensembl.org/) through the BioMart portal and appended to the *Bgh* DH14 genome (Spanu et al., 2010). Annotations were also downloaded from the Carbohydrate-Active enZymes Database (CAZy) ( http://www.cazy.org) and appended to the *Bgh* genome using *Fusarium graminearum* as a reference organism (Cuomo et al., 2007).

Genes that are potentially involved in cell wall polysaccharide biosynthesis were partitioned in two ways. The first method involved categorising the genes together based on their CAZy families. In the second method, the genes were segregated based on their KEGG predicted functions and EC numbers.

## Results

### Glycosidic linkage analysis of *Bgh* conidia

**Fig. 1:**
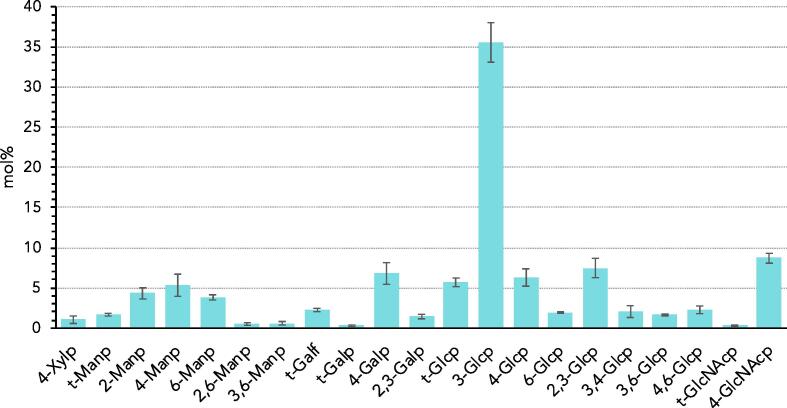
Glycosidic linkage analysis (mol%) of the carbohydrate fraction of the cell wall from ungerminated *Blumeria graminis* f. sp. *hordei* conidia. The different glycosidic linkages and pyranose or furanose forms of each monosaccharide were deduced from EI-MS spectra. Gal, galactose; Glc, glucose; GlcNAc, *N*-acetylglucosamine; Man, mannose; Xyl, xylose; ’p’ and ’f’ at the end of a monosaccharide abbreviation indicate that the residue occurs in the pyranose or furanose form, respectively; ’t-’ indicates a ’terminal’ monosaccharide that occurs at the non-reducing end of a glycan. Error bars indicate standard deviations calculated from three biological replicates.

Linkage analysis conducted on the *Bgh* conidia showed that a majority of the conidial cell wall polysaccharides consist of glucosyl residues (Glc) (63.1%) followed by mannosyl (Man) (16.2%), galactosyl (Gal) (10.7%), *N*-acetylglucosaminyl (GlcNAc) (9%) and xylosyl (1%) residues (Fig. 1). The linkage analysis revealed that the Glc fraction of the *Bgh* conidial cell wall was dominated by 1,3-linked Glc residues (56%) followed by 2,3-Glc (11.8%), 1,4-Glc (10%), and terminal Glc residues (9%). Almost all of the GlcNAc fraction of the cell wall consisted of 1,4-linked residues (97.7%), the remaining corresponded to terminal GlcNAc (3.3%). The comparatively low presence of terminal GlcNAc suggests that the chitin chains in the *Bgh* cell wall occur with a significantly high degree of polymerisation. Ten and a half percent of the Man fraction was found in the terminal form, followed by a relatively even distribution of 1,2-linked (26.5%), 1,4-linked (32.7%), and 1,6-linked (23.5%) Man residues. The majority of the Gal fraction in the cell wall was present in the pyranose form as 1,4-linked residues (64%) along with some 2,3-linked residues (13%). Both furanose (20.7%) and pyranose (2.8%) terminal forms were also present in the Gal fraction, with a significantly higher proportion of terminal Gal occurring in the furanose form. The trace amount of xylosyl residues detected in the cell wall polysaccharide fraction was present as 1,4-linked residues.

### Annotation of Carbohydrate Active enZymes (CAZy) in the *Bgh* genome

**Fig. 2:**
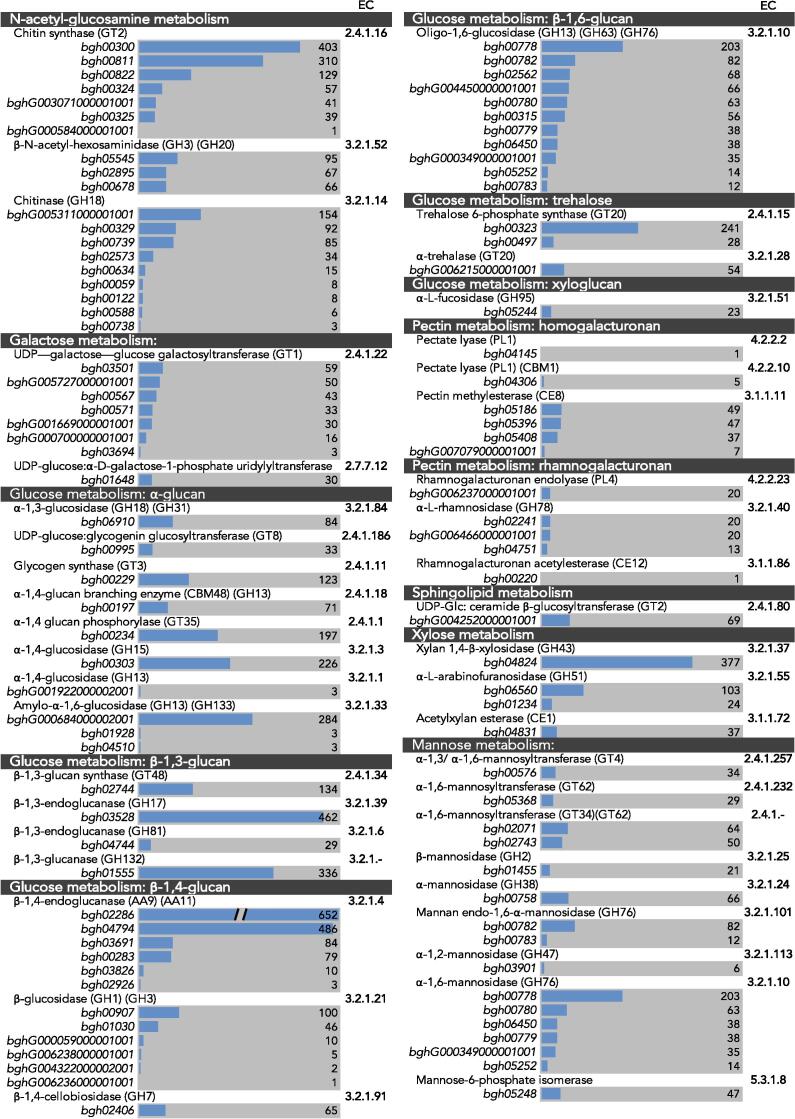

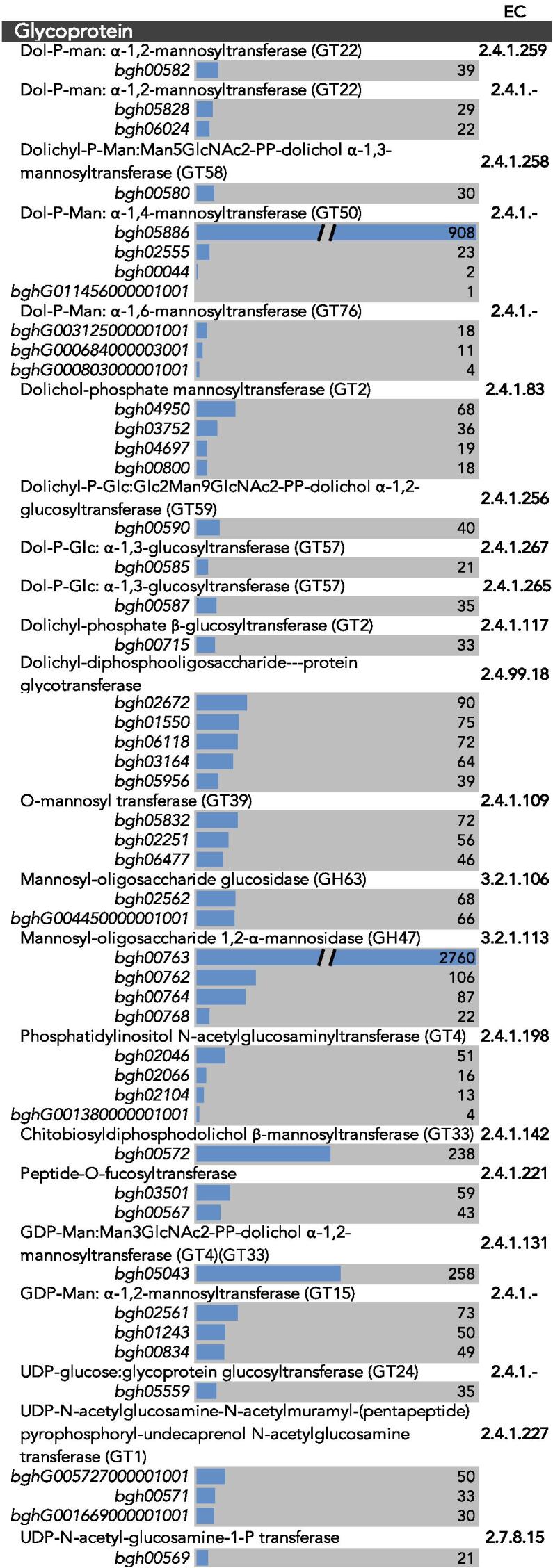
RNA-Seq transcript levels of genes involved in cell wall metabolism in the *Blumeria graminis* f. sp. *hordei* ungerminated conidia. Genes are organised by their KEGG predicted roles in cell wall metabolism. Transcript levels are displayed adjacent to the accession numbers of genes as RPKM values. Scale of the bars is 0–500 RPKM, with a few outliers exceeding 500. EC, Enzyme Commission numbers. (Continues on next page).

Genes that were potentially involved in the synthesis of *Bgh* cell wall polysaccharides were identified and annotated. The genome of *Bgh* DH14 (Spanu et al., 2010) was appended with KEGG, EC, PFAM, CAZy annotations and blasted against the annotated *F. graminearum* genome (Cuomo et al., 2007). Putative genes involved in cell wall biosynthesis were sorted based on their KEGG annotations and predicted functions (Fig. 2). However, as the KEGG classification does not cover all of the CAZy genes, a separate table of all annotated CAZy genes can be found in Supplementary Tables S2–S7. Three hundred and six CAZy genes were identified. These code for predicted auxiliary activity (AA) enzymes (25), carbohydrate-binding modules (CBM) (41), carbohydrate esterases (CE) (25), glycoside hydrolases (GH) (138), glycosyltransferases (GT) (93), and polysaccharide lyases (PL) (4) (Table 1).

**Table 1:** Copy number of *Blumeria graminis* f. sp. *hordei* Carbohydrate Active enZymes (CAZy) classified genes in each family. Auxiliary Activity (AA) enzymes, Carbohydrate Binding Modules (CBM), Carbohydrate Esterases (CE), Glycoside Hydrolases (GH), Glycosyl Transferases (GT) and Polysaccharide Lyases (PL).

CAZy genes	Number of genes
AA	25
CBM	41
CE	25
GH	138
GT	93
PL	4
Total	326

Multiple copies of putative chitin synthase genes (*Chs*) (GT2) were identified. Similar to *N. crassa*, seven copies of *Chs* genes were identified in *Bgh* (Sánchez-León et al., 2011), one for each chitin synthase class (*I-VII*). Naming of the *Bgh* chitin synthase genes was based on their class and similarity to *F. graminearum* chitin synthases.

A number of proteins involved in chitin modification were also identified, including three β-*N*-acetyl-hexosaminidases (GH3 and GH20), eleven chitinases (GH18 and CBM18), and four chitin deacetylases (CE4 and CBM18).

The *Bgh* genome encodes one functional copy of a putative β-1,3-glucan synthase (*Fks1*) (GT48). This trend is common in other filamentous fungi, in which a single (*Fks1*) gene product is potentially responsible for synthesising the entirety of the β-1,3-glucan in the fungal cell wall (Supplementary Table S6). The analysis did not uncover any gene that could be potentially involved in α-1,3-glucan biosynthesis. Nevertheless, genes that code for putative transglycosylating α-1,3-glucanosyltransferase enzymes (GH57) and α-1,3-glucosidases (GH18) are present within the genome.

Three putative β-1,3-glucanosyltransferases (GH72) were also identified (Supplementary Table S5). Enzymes affiliated with the GH72 family have been annotated as transglycosylases that are predicted to assist in either the formation of β-1,3-glucans with 1,6-branches or the elongation of β-1,3-glucan chains (Hartland et al., 1996, Mouyna et al., 1998). The presence of 1,3,6-linked glucosyl residues in the *Bgh* cell wall supports the possibility that the GH72 enzymes are involved in β-1,3-glucan branching. Two *Bgh* genes with similarity to the *S. cerevisiae KRE* genes, which have been suggested to be required for β-1,6-glucan synthesis (Kollár et al., 1997), were also present in the genome (Supplementary Table S5). These genes could also be acting in conjunction with candidate chitin β-1,6-glycanosyltransferases (*bgh02647* and *bghG001597000001001*) (Supplementary Table S5), which form cross-links between chitin and 1,3-glucans. These candidate genes encode GH16 transglycosylases that contain a CBM18 type of chitin-binding module. The presence of these genes, coupled with the presence of 1,6-linked glucans (Fig. 1), indicate that some β-1,6-glucans may form cross-links with β-1,3-glucans, chitin, and GPI-anchored oligosaccharides in the *Bgh* conidial cell wall. In addition to the anabolic enzymes, there are multiple hydrolytic enzymes involved in glucan metabolism. Three copies of exo-β-1,3-glucanases (GH5) were identified, along with four endo-β-1,3-glucanases (GH17 and GH81), and eight β-1,3-glucanases (GH16, GH55 and GH132) (Supplementary Table S5). A wide range of genes putatively involved in mannan biosynthesis were identified, which supports the presence of the various mannosyl linkages found in the cell wall analysis. A total of 31 mannosyltransferases were identified, many with redundant functions in glycoprotein metabolism. There were seven α-1,2-mannosyltransferases (GT4, GT15, GT22 and GT33), one α-1,3-mannosyltransferase (GT58), four α-1,4-mannosyltransferases (GT50), nine α-1,6-mannosyltransferases (GT32, GT34, GT62, GT76), one α-1,3/1,6-mannosyltransferase (GT4), six mannosyltransferases (GT2 and GT22), and three *O*-mannosyltransferases (GT39) (Fig. 2). As only a single GT4 was present, it is likely that it is responsible for the formation of the 1,3,6-linked mannosyl residues (Fig. 1). The hydrolytic enzymes that were predicted to act on mannose structures include one α-mannosidase (GH38), one β-mannosidase (GH2), seven α-1,2-mannosidases (GH47 and GH92), six α-1,6-mannosidases (GH76), and three endo α-1,6-mannosidases (GH76) (Fig. 2).

While a large number of genes were assigned to the metabolism of the major cell wall polysaccharides, a more limited pool of genes were identified to be putatively involved in the metabolism of galactose and xylose containing polysaccharides. A few candidates involved in the UDP-galactose pathway were identified (GT1) along with two galactosyltransferases (GT34) that are potentially involved in the transfer of galactosyl residues to acceptor molecules. Although there were no strong candidates identified for the incorporation of xylosyl residues in the *Bgh* cell wall polysaccharides, genes encoding the xylan hydrolytic enzymes β-1,4-xylosidase (GH3 and GH43) and acetylxylan esterase (CE1) were found within the genome. The lack of any candidate genes for the incorporation of xylosyl residues into other polysaccharide polymers is resultant from the KEGG classification system, which depends on previously characterised orthologues in other species. As there is a lack of characterisation of xylose metabolism genes in fungal species, the KEGG database is insufficient for identifying target genes from this class. Based on the predicted functions of the CAZy classes, candidate xylosyltransferases may be found in the GT1, GT4 and GT90 families.

### Transcriptome analysis of ungerminated *Bgh* conidia

The majority of the candidate cell wall genes (Fig. 2 and Supplementary Table S8) were expressed at detectable levels in the RNA-Seq data from the ungerminated *Bgh* conidia. Only the genes that exhibited detectable transcripts in the KEGG sorted list are shown in Fig. 2, along with the corresponding accession numbers. Supplementary Table S8 displays all the CAZy annotated genes along with their corresponding transcript levels. To validate the RNA-Seq dataset, qPCR was conducted on a subset of cell wall related genes, chiefly GHs involved in chitin and glucan metabolism (Supplementary Figs. S1 and S2). A good correlation was observed between the RNA-Seq and qPCR expression profiles ($y=429.14x-18215\text{;}R^{2}=0.93$), indicating the reliability of the RNA-Seq dataset (Supplementary Fig. S3).

All of the seven *Chs* genes are expressed in the ungerminated conidia (Fig. 2). *Chs1* (*bgh00300*) displayed the highest expression, which is not unexpected as class I chitin synthases are generally expressed at high levels during vegetative growth (Roncero, 2002). *Chs7* (*bgh00811*) exhibited the second highest rate of expression, followed by *Chs3* (*bgh00822*). *Chs5* (*bgh00324*) and *Chs6* (*bgh00325*) showed low levels of expression in the conidia.

Transcripts of *Chs2* (*bghG000584000001001*) and *Chs4* (*bghG003071000001001*) showed the lowest levels of expression in the conidia. This could be because in other fungal species these two classes of CHS are generally only active during mating and sporulation. Class II chitin synthases in particular are associated with septum formation, while class IV chitin synthases are associated with spore maturation (Roncero, 2002). The chitinases generally displayed low levels of expression, however one chitin deacetylase (*bgh06022*) showed considerably high transcript levels, 670 RPKM (Supplementary Table S8). Chitin deacetylases catalyse post-synthetic modifications to chitin by partially or fully deacetylating chitin to form chitosan. As chitosan is a poor elicitor of the plant immune response and resistant to plant chitinases, the ungerminated *Bgh* may be altering the structure of chitin in preparation for pathogenesis (Hadwiger, 2013).

The *Fks1* (*bgh02744*) transcript displays a relatively moderate level of expression (134 RPKM) (Fig. 2), yet a number of enzymes involved in glucan remodelling are highly expressed in the ungerminated conidia. These include two of the β-1,3-glucanosyltransferases (GH72) (*bgh00776*, and *bgh00774*) (Supplementary Table S8), an exo-β-1-3-glucanase (GH5) (*bgh00086*) (Supplementary Table S8), a β-1,3-endoglucanase (GH17) (*bgh03528*) (Fig. 2) and a β-1,3-glucanase (GH16 (*bgh00726*) and GH132 (*bgh01555*)) (Supplementary Table S8 and Fig. 2, respectively). The β-1–3-glucanosyltransferase gene (*bgh00776*) in particular was one of the highest expressed (1185 RPKM). The two GH72 β-1,3-glucanosyltransferase genes (*bgh00776* and *bgh00774*) are similar to the *A. fumigatus GEL1* and *GEL7* genes, respectively. *GEL1* was found to be highly expressed during mycelial growth and was important in virulence while *GEL7* is implicated in conidiation (Mouyna et al., 2005, Gastebois et al., 2010, Zhao et al., 2014). The elevated transcript levels of the various enzymes involved in glucan metabolism may suggest that a high degree of β-1,3-glucan remodelling occurs in the *Bgh* conidia. Several genes involved in *N*-glycan biosynthesis and modification of glycan side chains are also highly expressed, including an α-1,2-mannosidase (GH47) (*bgh00763*), a Dol-P-Man:α-1,4-mannosyltransferase (GT50) (*bgh05886*) and a β-1,4-xylosidase (GH43) (Fig. 2). In fact, the gene encoding for the α-1,2-mannosidase is the highest expressed gene in the conidia (2760 RPKM). Chitobiosyldiphosphodolichol β-mannosyltransferase (GT33) (*bgh00572*) and GDP-Man:Man_3_GlcNAc_2_-PP-dolichol α-1,2-mannosyltransferase (GT4) (GT33) (*bgh05043*), which are also involved in *N*-glycan biosynthesis, exhibited moderate levels of expression. Given that the conidia were collected from developing conidiophores, it is not unexpected to see a significant level of gene expression involved in the maintenance of the cell wall and final alterations prior to release.

## Discussion

A comparison of the *Bgh* cell wall against other well characterised species, such as *N. crassa* (Mélida et al., 2015), *A. fumigatus* (Fontaine et al., 2000), *S. cerevisiae* (Fleet, 1985), and *Rhynchosporium secalis* (Pettolino et al., 2009), demonstrates the contrasting composition of the *Bgh* cell wall. Despite the significant structural role of chitin, especially in filamentous fungi, this polysaccharide only represents 9% of the total cell wall in the *Bgh* conidia. The chitin deacetylase (*bgh06022*) showed high transcript levels (Supplementary Table S8) in the conidia. This could infer that a majority of the chitin in the conidia cell wall is in a deacetylated state, possibly to reduce the opportunity in eliciting the plant immune response. The relative decrease of chitin in the cell wall compared to other fungi is compensated by an increase in glucan content (63.1%). The prevailing form of glucan in the cell wall is 1,3-linked (56%), however, the distribution of alpha and beta linkages is unknown, with both *Fks1* and candidate α-1,3-glucanosyltransferases displaying relatively moderate expression levels in the conidia (Fig. 2).

Chitin, β-1,3-glucans, and glycoproteins are cross-linked by β-1,6-glucans in *S. cerevisiae* and *C. albicans* (Kollár et al., 1995, Kollár et al., 1997, Netea et al., 2008). The presence of 1,6 (2%) and 1,3,6-linked (1.6%) residues in the *Bgh* cell wall suggests that β-1,3-glucans, chitin, and glycoproteins are cross-linked by β-1,6-glucans. This is further supported by the presence of putative β-1,3-glucanosyltransferases (GH72) and candidate chitin β-1,6-glycanosyltransferases. Not all fungi implement the use of β-1,6-glucan cross-links. For example, species such as *N. crassa*, *R. secalis* and *A. fumigatus*, have extremely low or no detectable amounts of 1,6-linked glucan in their cell walls (Table 2), indicating that there may be alternate means of cross-linking cell wall components for structural integrity.

**Table 2:** Permethylation glycosidic linkage analysis (mol%) of the carbohydrate fraction of the total cell walls from ungerminated *Blumeria graminis* f. sp. *hordei*, *Neurospora crassa* (Mélida et al., 2015), *Rhynchosporium secalis* (Pettolino et al., 2009), *Aspergillus fumigatus* (Fontaine et al., 2000) (Alcohol insoluble fraction only) and *Saccharomyces cerevisiae*. The different glycosidic linkages and pyranose or furanose forms of each monosaccharide were deduced from EI-MS spectra. Sugar derivatives nomenclature used: Gal, galactose; GalNAc, *N*-acetylgalactosamine; Glc, glucose; GlcA, glucuronic acid; GlcNAc, *N*-acetylglucosamine; Man, mannose; Rha, rhamnose; Xyl, xylose; ’p’ and ’f’ at the end of a monosaccharide abbreviation indicate that the residue occurs in the pyranose or furanose form, respectively; ‘t-’ indicates a ‘terminal’ monosaccharide that occurs at the non-reducing end of a glycan.

Linkage	*Bgh*	*N. crassa*	*R. secalis*	*A. fumigatus*	*S. cerevisiae*
t-Gal*f*	2.2	1.0		2.2	
5-Gal*f*			12.0	8.1	
t-Gal*p*	0.3	3.0	2.5		0.2
4-Gal*p*	6.8				
2,3-Gal*p*	1.4				0.4
2,3,4,6-Gal*p*			0.1		
4-GalNAc*p*				3.9	
t-Glc*p*	5.7	13.0	13.9	0.3	5.5
2-Glc*p*			0.1		
3-Glc*p*	35.6	54.0	11.6	43.3	26.9
4-Glc*p*	6.3	4.0	17.2	14.2	
6-Glc*p*	2.0		0.5	0.3	16.0
2,3-Glc*p*	7.5		1.5		7.0
3,4-Glc*p*	2.1		0.5	0.3	0.7
3,6-Glc*p*	1.6	5.0	2.7	1.6	2.0
4,6-Glc*p*	2.3		0.5		
2,3,4-Glc*p*			0.1		
3,4,6-Glc*p*			0.1		
2,3,4,6-Glc*p*			2.3		
t-GlcNAc*p*	0.3	0.5			0.1
4-GlcNAc*p*	8.7	10.0	6.3	17.65	0.6
t-Man*p*	1.7	1.5	3.1	0.1	15.7
2-Man*p*	4.3	3.0	4.3	2.4	7.9
3-Man*p*			2.9		4.0
4-Man*p*	5.3				0.3
6-Man*p*	3.8		4.4	1.2	1.2
2,3-Man*p*			0.9		
2,4-Man*p*			0.1		
2,6-Man*p*	0.5	4.0	2.4	1.3	10.2
3,6-Man*p*	0.6		0.6		0.4
4,6-Man*p*				0.5	
2,4,6-Man*p*			0.8		
2,3,4,6-Man*p*			0.6		
t-Rha*p*			5.7		
2-Rha*p*			1.3		
2,3,4,6-Rha*p*			0.1		
4-Xyl*p*	1.0		0.1		
Total	100.0	99.0	99.2	97.4	100.1

In comparison to the other fungi, the total proportion of mannosyl residues in the *Bgh* cell wall is similar to that of *R. secalis*, though the distributions of mannosyl linkages vary considerably (Table 2). *Bgh* has the highest amount of 1,4-linked mannosyl residues compared to the other fungi. Chitobiosyldiphosphodolichol β-mannosyltransferase (GT33) (*bgh00572*) and a β-1,4-mannosyltransferase, displayed moderate expression in the conidia (238 RPKM), while Dol-P-Man:α-1,4-mannosyltransferase (GT50) (*bgh05886*) was highly expressed (908 RPKM). In addition, α-1,2-mannosyltransferase (*bgh05043*) also exhibited moderate expression and the α-1,2-mannosidase (GH47) gene (*bgh00763*) was one of the highest expressed (Fig. 2). These genes are all predicted to be involved in the synthesis of *N*-glycans and may imply that mannan chains are modified during the conidial stage.

The galactose component of the *Bgh* cell wall polysaccharides is primarily composed of 1,4-linked galactopyranosyl residues (63.6%), which is seldom present in fungal cell walls. There has only been scarce mentions of galactopyranosyl residues in fungal cell wall analysis studies. Though trace amounts of 1,4-linked galactopyranosyl residues have been detected in *Magnaporthe grisea* (>1%) (Samalova et al., 2016) and galactopyranosyl residues have been detected in *R. secalis* (Pettolino et al., 2009), these composed a minute portion of the cell wall compared to *Bgh*.

Unlike genes related to the other cell wall polysaccharides, only a few candidate genes involved in galactose metabolism could be identified. The few genes that were identified displayed relatively low levels of expression in the conidia (Fig. 2). In *Aspergillus*, UDP-galactopyranose mutase converts UDP-galactopyranose to UDP-galactofuranose for the biosynthesis of galactofuranose glycoconjugates. Galactofuranose is an important component of fungal cell wall polysaccharides that is not found in any mammals or higher plants and has been shown to play an important role in virulence and cell wall structure in *Aspergillus nidulans* and *A. fumigatus* (El-Ganiny et al., 2008, Schmalhorst et al., 2008, Afroz et al., 2011). The diminished presence of galactofuranosyl residues in the *Bgh* conidial cell wall could have evolved to avoid eliciting the plant immune response. As an obligate biotroph, the survival strategy of *Bgh* is to remain undetected and not harm the host.

Interestingly, 1,4-linked xylosyl residues were detected in the *Bgh* conidial cell wall. In the past, a hallmark of ascomycetes was the absence of xylosyl residues in the cell wall. This linkage type, however, has previously been found in basidiomycetes such as *Tremella mesenterica, Ustilago maydis* and *Crytococcus neroformans* (Reid and Bartnicki-Garcia, 1976, Ruiz-Herrera et al., 1996, Park et al., 2012). Trace amounts of xylosyl residues have recently been recorded in ascomycetes, such as *M. grisea* (Samalova et al., 2016). Little is known about the role of xylose in the fungal cell walls. It could potentially be a component of xylomannan, which has been detected in the cell walls of *Flammulina velutipes* and *Trichosporon cutaneum* (Depree et al., 1993, Kawahara et al., 2016) and is found in diverse taxa, ranging from plants, insects, and amphibians (Walters et al., 2011). Xylomannan has been proposed to act as an antifreeze molecule that enables organisms to overwinter.

Not enough is known about xylose metabolism in fungi to be able to select strong candidate xylosyltransferase genes from the genome, although the candidate pool may be narrowed down by looking at the predicted functions of various CAZy classes. Only a small number of genes thought to be involved in xylose metabolism were identified, with potential CAZy classes including GT1, GT4, GT34, and GT90, which all have predicted xylosyltransferase functions. Knowledge of the *Bgh* cell wall composition is the first step in understanding its structure. Further experimental evidence will be required to determine how each linkage type is incorporated into individual polysaccharides and how these are organised within the cell wall.

Plants possess an innate immune system that is able to identify invading pathogens by recognising evolutionary conserved pathogen or microbial associated molecular patterns (PAMPs or MAMPs) through pattern recognition receptors (PRR) (Ferrari et al., 2013). Major cell wall components, such as chitin and β-glucans, are strong elicitors and activate PAMP-triggered immunity (PTI). The diverse compositions observed in the fungal cell wall glycoproteins found in the outer cell wall layers of different species may serve to mask the more widespread immunoreactive cell wall components. A common strategy to evade host detection involves modifying the cell wall. Relative to chitin and β-glucan, chitosan and α-glucans are much weaker inducers of the defense responses in most plants (Fujikawa et al., 2012, Hadwiger, 2013). By deacetylating chitin to chitosan or accumulating α-1,3-glucan on the outer surface of the cell wall, pathogens have a much greater chance of evading detection. As an obligate biotroph *Bgh* would aim to remain undetected to avoid triggering the PTI. It would stand to reason that the *Bgh* cell wall would reflect this lifestyle as well. With the high expression of chitin deacetylase, it is likely that the majority of the chitin present in the conidial cell wall has been deacetylated to chitosan. The glycoproteins would be cross-linked to the β-1,3-glucan core through β-1,6-glucans and decorated with galactofuranosyl residues and xylomannan side chains that are rarely present in fungal cell walls. Considering that 1,3-linked glucosyl residues were most abundant in the *Bgh* conidial cell wall, it is also possible that α-1,3-glucans are decorating the exterior of the *Bgh* cell wall as well. The design of the cell wall would conceal the *Bgh* pathogen with chitosan and rare polysaccharides so that it can live an epiphytic lifestyle without alerting the host.

In the *Bgh* genome, there is a notable reduction in genes that encode enzymes involved in the degradation of the plant cell wall. Genome annotations of other obligate biotrophic pathogens, such as corn smut (*U. maydis*) and rust fungi (*Melampsora laricipopulina* and *Puccinia graminis* f. sp. *tritici*), also revealed diminished numbers in cell wall degrading enzymes (Kämper et al., 2006, Duplessis et al., 2011). In comparison with hemibiotrophic and necrotrophic fungi, biotrophic fungi have the smallest array of CAZy genes (Raffaele and Kamoun, 2012, Zhao et al., 2013). The loss of these genes are mirrored in the genomes of numerous unrelated biotrophic fungi and oomycetes, indicating convergent adaptations to the obligate biotrophic lifestyle on plants (Raffaele and Kamoun, 2012). The CAZy annotations of the *Bgh* genome show that there have been expansions and reductions within gene families compared to other well characterised fungal species (Supplementary Tables S2–S7).

Prior analyses of the *Bgh* genome have concluded that *Bgh* had an extreme reduction in enzymes devoted to degrading the plant cell walls (Spanu et al., 2010, de Witt et al., 2012, Hacquard et al., 2013). However, although the number of cell wall degrading genes are diminished, the original analysis had underestimated the *Bgh* CAZy gene numbers. Spanu et al. (2010) did not identify any candidate cellulose, xylan or pectin degrading enzymes and they identified three GH families only (GH16, GH61 and GH81), while de Witt et al. (2012) identified 63 GH genes and a few genes in the CE and CBM families. This is contrary to our data, as we have identified a substantially greater number of genes in all the CAZy families using a combination of CAZy annotations, PFAM, and BLAST searches (Supplementary Tables S2–S7). Although diminished compared to other hemibiotrophic and necrotrophic fungi, *Bgh* does possess a significant arsenal of cell wall degrading and modifying enzymes for pathogenesis and cell wall modification.

The largest reduction in gene numbers can be seen with the GH families, especially when compared to *A. fumigatus* and the hemibiotrophic plant pathogen *R. secalis*. Many of the GH families in *Bgh* showed decreased gene numbers or absence of genes compared to the other fungi, with the most dramatic reductions seen in the GH2, GH3 and GH43 families (Supplementary Tables S5). The GH2 family is reported to contain β-mannosidase, β-galactosidase, exoglucosaminidase, and chitosanase activities. The GH3 family contains β-xylosidases and β-glucosidases and the GH43 family contains endo-α-1,5-arabinanase, α-arabinofuranosidase, and β-xylosidase activities.

There was also a reduction within the CE1 and CE5 families, which are associated with acetylxylan esterase and cutinase functions (Supplementary Table S4). In addition, there was a significant reduction in the AA3 and AA9 families. The AA families contain redox enzymes that act in conjunction with hydrolytic CAZy enzymes. The AA3 family mostly contains cellobiose dehydrogenases (Levasseur et al., 2013) while AA9 enzymes have been shown to act as lytic polysaccharide monooxygenases (LPMO), working synergistically with cellulases in cellulose hydrolysis (Kim et al., 2015). While the total number of genes within the GTs was comparable to all other species there were expansions in GT32, GT33, GT50, and GT76, which are all families associated with mannosyl and galactosyltransferase activities.

The CBMs had notable increases in the CBM1, CBM18, and CBM50 families, which have been demonstrated to bind to cellulose (CBM1) and chitin (CBM18 and CBM50) (Mello and Polikarpov, 2014, Akcapinar and Kappel, 2015, Liu and Stajich, 2015). Expansions in CBMs have been associated with pathogenicity in various fungi, by playing a role in the resistance of the pathogen to the host defence response (Abramyan and Stajich, 2012, Amselem et al., 2011). The increase in chitin-binding modules may assist *Bgh* in dampening chitin-induced PTI. Plants release chitinases as a general protective measure against fungal pathogens and are able to recognise chitin oligomers. To avoid chitin-induced PTI, fungi such as *Cladosporium fulvum* secrete the evolutionary conserved LysM (CBM50) domain containing extracellular effector protein 6 (Ecp6) during infection, which sequesters the chitin fragments and outcompetes the PRRs to avoid detection (de Jonge et al., 2010). To guard against chitinases, *C. fulvum* also secretes the chitin-binding effector Avr4 (CBM14), which binds to the chitin present in the fungal cell wall to protect it from degradation (van den Burg et al., 2006). It was recently demonstrated that secreted proteins containing a CBM18 also have the capacity to bind to chitin in the cell wall and protect fungi from chitinases (Liu and Stajich, 2015). The expansion of CBM18 has also been observed in other pathogenic fungi (Abramyan and Stajich, 2012, Amselem et al., 2011).

The genomic analysis revealed a comprehensive list of CAZy families involved in polysaccharide metabolism and supports previous speculations of *Bgh* possessing an extensively reduced set of CAZy genes due to its nature as an obligate biotroph. Further analysis demonstrated an expansion of the CBM families, not identified in previous investigations and classified substantially more CAZy genes than were previously thought to be present in the *Bgh* genome.

The conidia are generally considered metabolically inactive structures, developing on the conidiophores and remaining dormant until triggered to germinate. Contrary to this viewpoint, recent studies on dormant conidia have demonstrated that they are metabolically active (Novodvorska et al., 2016). Consistent with this observation, our transcriptome analysis reveals that a multitude of genes associated with polysaccharide metabolism are active in the *Bgh* conidia. The conidial cell wall, while not experiencing any active biosynthesis requirements due to growth, is most likely undergoing many reconstruction and maintenance processes, with the cell wall composition and transcriptome data suggesting that the conidial cell wall was modified to conceal itself from the plant host.

The transcriptome and genome annotations of the *Bgh* cell wall synthetic and hydrolytic machinery will be a useful resource for locating candidate genes involved in polysaccharide metabolism for further analysis to investigate their role in cell wall biosynthesis. While knockouts are not a feasible option yet for *Bgh*, it is possible to perform gene knockdowns using host-induced gene silencing (HIGS) to silence target cell wall genes and determine their role in cell wall biosynthesis, growth, and survival (Nowara et al., 2010, Pliego et al., 2013). Further investigations should be conducted on the cell wall composition during various stages of *Bgh* pathogenesis in the future. In addition to major cell wall elements, the subtle species-specific differences in the cell wall structures and composition may play a significant part in supporting cell wall integrity and could be ideal targets for disease control.

## Author’s contributions

Conceived and designed the experiments: TP VB AL. Performed the experiments: TP JS NS XX. Analyzed the data: TP JS NS XX VB AL. Contributed reagents/materials/analysis tools: VB. Wrote and edited the paper: TP AL VB.

## Funding

This work was supported by the Australian Research Council Centre of Excellence in Plant Cell Walls (CE110001007).

## Declaration of Competing Interest

The authors declare that they have no known competing financial interests or personal relationships that could have appeared to influence the work reported in this paper.

## Supplementary data

Supplementary Fig. S1 Comparison of chitin synthase RNA-Seq (RPKM) and qPCR (normalised arbitrary units) transcript levels in *Blumeria graminis* f. sp. *hordei* conidia.

Supplementary Fig. S2 Comparison of RNA-Seq (RPKM) and qPCR (normalised arbitrary units) transcript levels in *Blumeria graminis* f. sp. *hordei* conidia of various genes implicated in glucan metabolism.

Supplementary Fig. S3 Correlation between RNA-Seq (RPKM) and qPCR (normalised arbitrary units) from *Blumeria graminis* f. sp. *hordei* conidia. Genes chosen were GHs involved in chitin and glucan metabolism.

Supplementary Table S1 Oligonucleotides used for qPCR of *Blumeria graminis* f. sp. *hordei* cell wall related genes.

Supplementary Tables S2–S7 **Table S2**. Copy number of genes classified in CAZy Auxiliary Activity (AA) modules from *Blumeria graminis* f. sp. *hordei*, *Neurospora crassa*, *Rhynchosporium secalis*, *Aspergillus fumigatus* and *Saccharomyces cerevisiae*. **Table S3**. Copy number of genes classified in CAZy Carbohydrate Binding Modules (CBM) from *Blumeria graminis* f. sp. *hordei*, *Neurospora crassa*, *Rhynchosporium secalis*, *Aspergillus fumigatus* and *Saccharomyces cerevisiae*. **Table S4**. Copy number of genes classified in CAZy Carbohydrate Esterase (CE) modules from *Blumeria graminis* f. sp. *hordei*, *Neurospora crassa*, *Rhynchosporium secalis*, *Aspergillus fumigatus* and *Saccharomyces cerevisiae*. **Table S5**. Copy number of genes classified in CAZy Glycoside Hydrolase (GH) modules from *Blumeria graminis* f. sp. *hordei*, *Neurospora crassa*, *Rhynchosporium secalis*, *Aspergillus fumigatus* and *Saccharomyces cerevisiae*. **Table S6**. Copy number of genes classified in CAZy Glycosyl Transferase (GT) modules from *Blumeria graminis* f. sp. *hordei*, *Neurospora crassa*, *Rhynchosporium secalis*, *Aspergillus fumigatus* and *Saccharomyces cerevisiae*. **Table S7**. Copy number of genes classified in CAZy Polysaccharide Lyase (PL) modules from *Blumeria graminis* f. sp. *hordei*, *Neurospora crassa*, *Rhynchosporium secalis*, *Aspergillus fumigatus* and *Saccharomyces cerevisiae*.

Supplementary Table S8 RNA-Seq transcripts of all CAZy groups in ungerminated *Blumeria graminis* f. sp. *hordei*. Transcripts are displayed as RPKM values.
