# Supplementary figures and images for: Composition and biosynthetic machinery of the Blumeria graminis f. sp. hordei conidia cell wall
Source: Cell Surf. 2019 Aug 14;5:100029. doi: 10.1016/j.tcsw.2019.100029 (PMC7388969; doi:10.1016/j.tcsw.2019.100029)

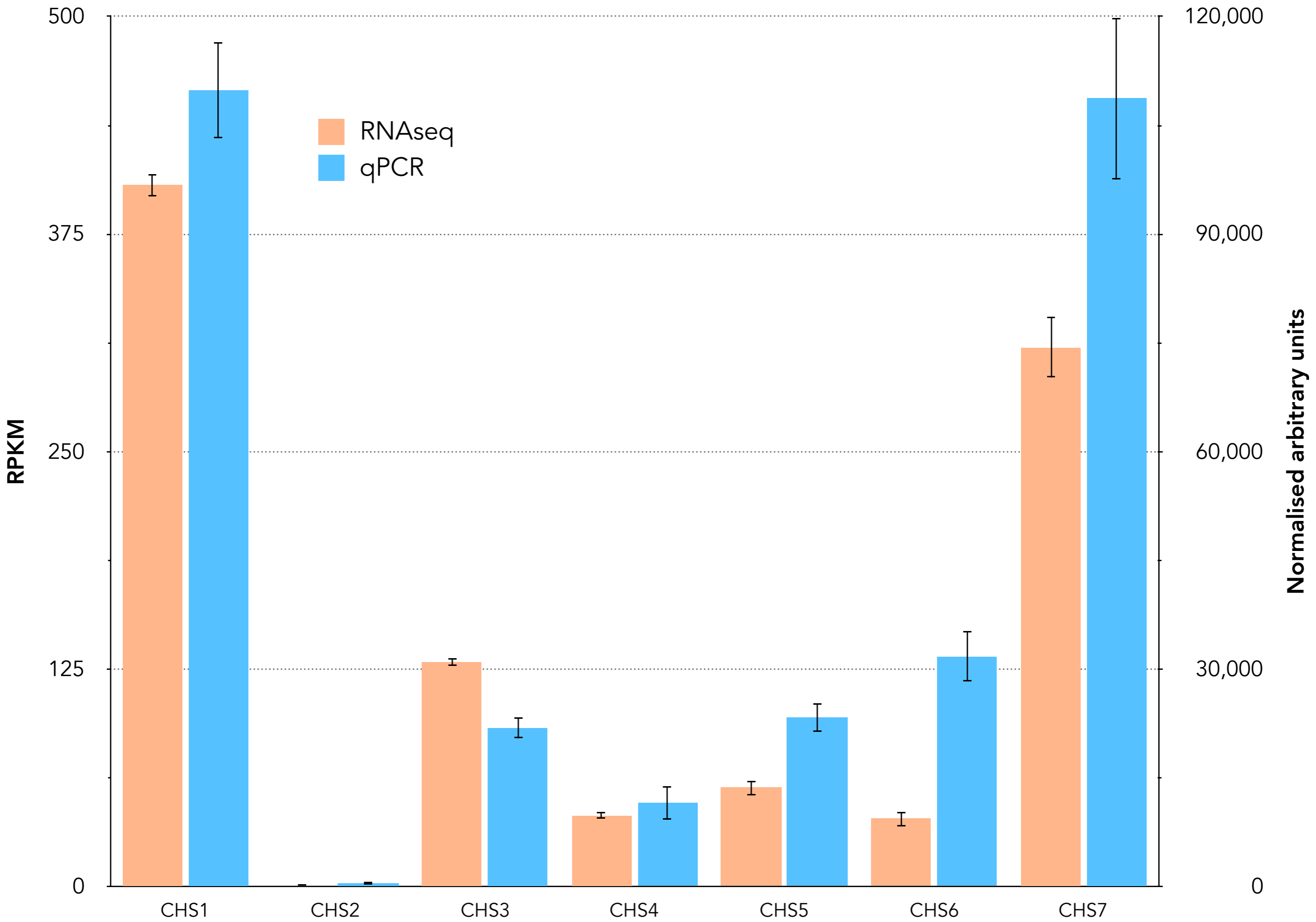

Supplement: Supplementary Fig. S1 — Comparison of chitin synthase RNA-Seq (RPKM) and qPCR (normalised arbitrary units) transcript levels in Blumeria graminis f. sp. hordei conidia. [file mmc1.pdf]

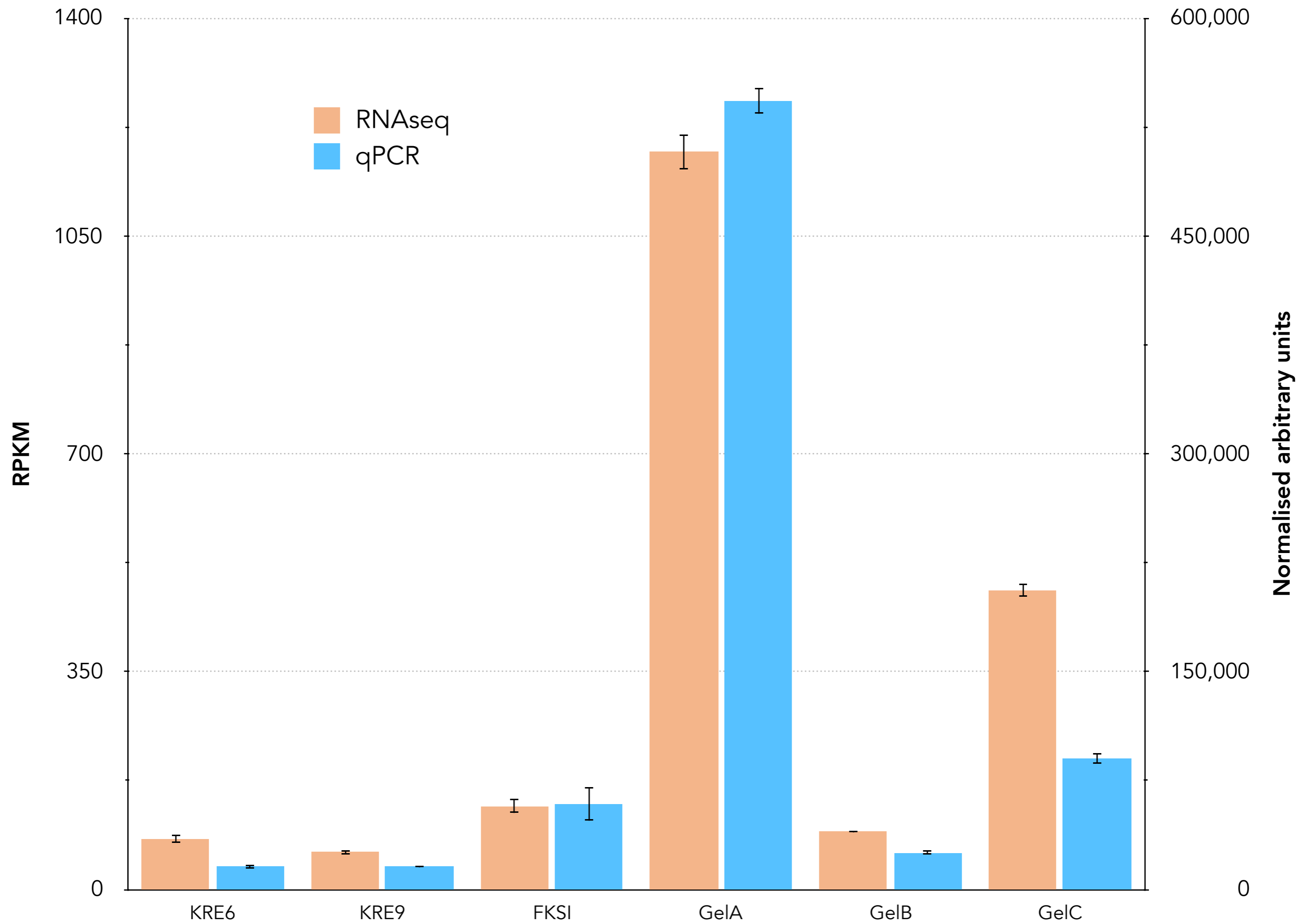

Supplement: Supplementary Fig. S2 — Comparison of RNA-Seq (RPKM) and qPCR (normalised arbitrary units) transcript levels in Blumeria graminis f. sp. hordei conidia of various genes implicated in glucan metabolism. [file mmc2.pdf]

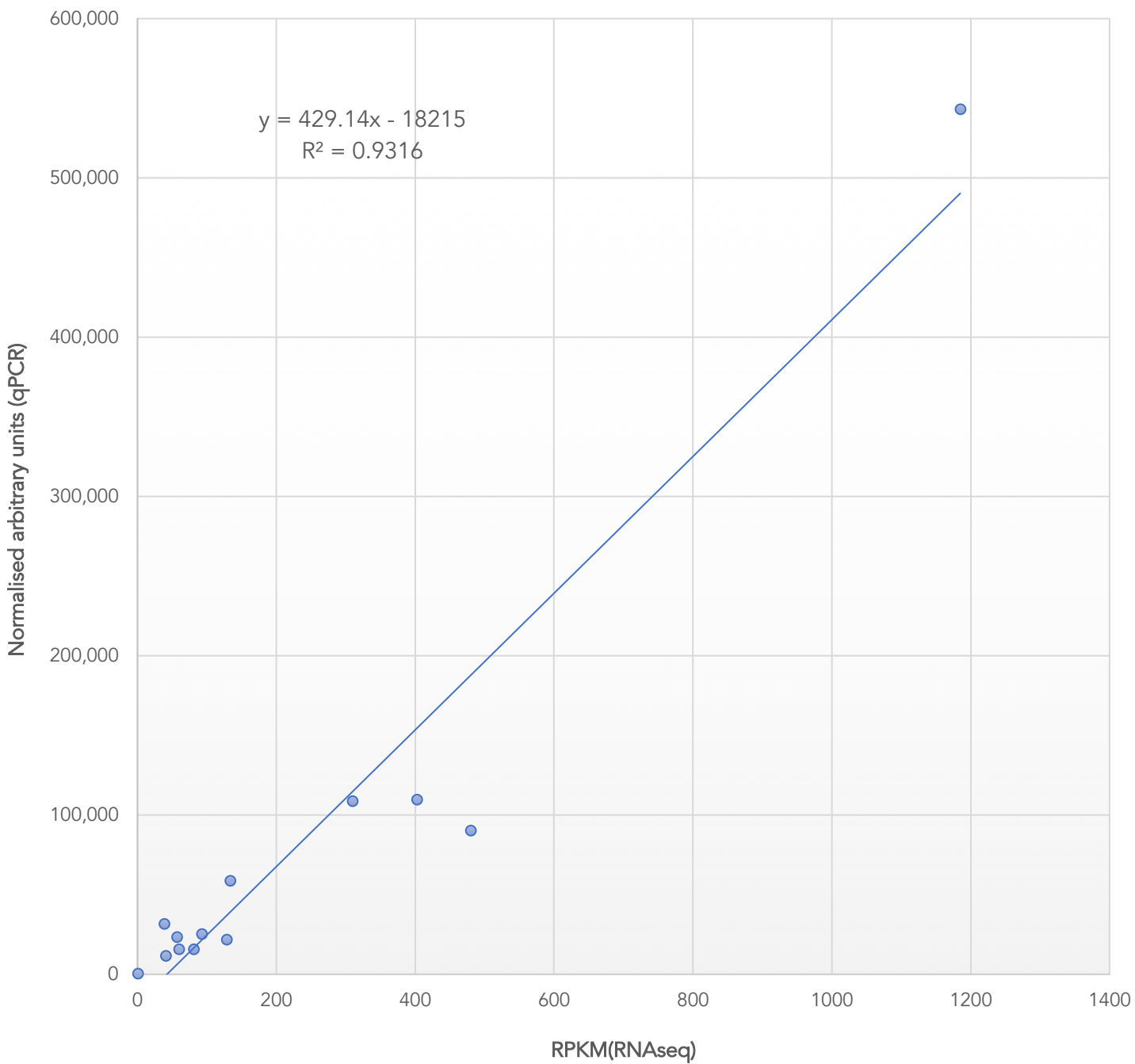

Supplement: Supplementary Fig. S3 — Correlation between RNA-Seq (RPKM) and qPCR (normalised arbitrary units) from Blumeria graminis f. sp. hordei conidia. Genes chosen were GHs involved in chitin and glucan metabolism. [file mmc3.pdf]
